# Clinical characteristics of liver failure with hemophagocytic lymphohistiocytosis

**DOI:** 10.1038/s41598-019-43909-w

**Published:** 2019-05-31

**Authors:** Jinling Dong, Fang Xie, Lin Jia, Juan Li, Zhongjie Hu, Yueke Zhu, Hongwei Yu, Yujuan Zhao, Qinwei Yao, Qinghua Meng

**Affiliations:** 1 Department of Severe Liver Disease Medical Center, Beijing You An Hospital, Capital Medical University, Beijing, China; 2 Department of Cardiology, The First Affiliated Hospital of Harbin Medical University, Harbin, China

**Keywords:** Liver diseases, Hepatitis

## Abstract

Liver failure with hemophagocytic lymphohistiocytosis (HLH) is a life-threatening syndrome with high mortality. The aim of this study was to decipher clinical and laboratory characteristics of hemophagocytic lymphohistiocytosis after definite diagnosis of liver failure and to provide clues for early diagnosis and treatment of HLH in patients with liver failure. Eleven patients diagnosed with liver failure and HLH were retrospectively investigated in this study. All patients presented with jaundice, persistent high-grade fever, pancytopenia, splenomegaly, evidence of hemophagocytes in the bone marrow and laboratory abnormalities indicating HLH. The average interval from the earliest diagnosis of liver failure to a definitive diagnosis of HLH was 17.27 days. Six (54.55%) patients died during follow-up. For patients with liver failure after admission and subsequently definitively diagnosed with HLH, bilirubin and INR were significantly decreased. HLH is definitely diagnosed at an intermediate or late stage when patients have already suffered from liver failure. The initial dose of glucocorticoid (methylprednisolone) was decreased to 1–1.5 mg/kg/d and gradually reduced thereafter. In conclusion, for patients with liver failure, HLH should be screened as early as possible upon persistent fever, splenomegaly and unexplained pancytopenia. For patients with liver failure and HLH, the dosage of glucocorticoid should be reduced to avoid serious side effects.

## Introduction

Liver failure is a life-threatening syndrome with high mortality characterized by hepatocellular necrosis. This serious liver injury is caused by a variety of factors, such as viral infections, hepatotoxic drugs or immune-mediated attack. It can develop without antecedent liver diseases or from acute-on-chronic liver diseases. Liver failure may lead to disorders of synthesis, detoxification, excretion and biotransformation function or severe decompensation. This is a group of clinical syndromes, including coagulation disorders, jaundice, hepatic encephalopathy and ascites^1^. HLH is a rare clinical syndrome characterized by an uncontrolled and ineffective hyperinflammatory response^2^ with an all-cause mortality rate of 75% as reported in large series^3^. The clinical course of HLH usually progresses to “cytokine storm” due to uncontrolled macrophage activity and hypersecretion of cytokines^4^. Primary or familial HLH occurs most often in the pediatric age group, with approximately 50% of cases attributed to PFR1 gene mutations, resulting in deficient perforin^5^.

Secondary HLH is common in adults and involves numerous etiologies, including all types of infections, malignancies, and Immune diseases^6^. Approximately 85% of adult patients had abnormal liver function^7^. Secondary HLH is associated with multiorgan failure, with high rates of morbidity and mortality^8^. Especially, acute liver failure caused by HLH has a high mortality^9^. However, the incidence of liver failure combined with HLH is extremely rare. The coexistence of two clinical conditions likely delays diagnosis, leading to rapid disease progression and substantially increased mortality. Although an optimal treatment of HLH is still being debated, the current treatment of HPS is mainly based on the HLH-2004 regimen^10^. Glucocorticoids play an important role in the treatment of liver failure with HLH, however, an optimal dose beneficial to control disease is worth exploring. Many gastroenterologists and hepatologists lack knowledge of and experience in early diagnosis and treatment of liver failure with HLH. Therefore, we retrospectively studied medical records of eleven patients diagnosed with liver failure and HLH, to demonstrate clinical and laboratory characteristics and to provide clues for early diagnosis and treatment of this fatal disease.

## Patient and Method

**Figure 1:**
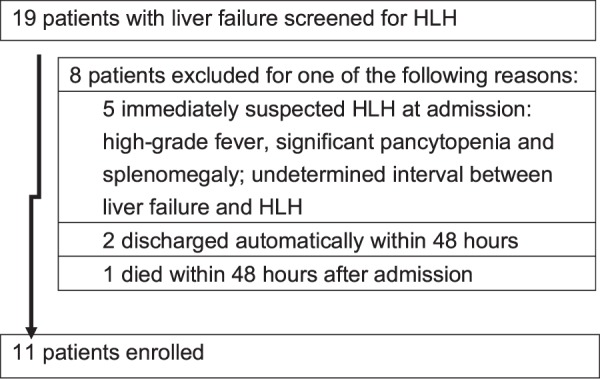
Eleven liver failure patients with HLH were identified in the study: Eleven Patients were included in statistical analysis of data in this study of: (1) met the 2012 Chinese Guideline Criteria for diagnosis and treatment of liver failure 1; (2) did not meet the criteria for diagnosis of HLH (HLH-2004)^10^ at admission but met the criteria during the course of treatment.

The study was conducted according to the Helsinki Declaration and was approved by Institutional Review Board of Beijing You-An Hospital Affiliated Capital Medical University, Beijing, China. The study has obtained written informed consent from each patient or patient’s authorized family member. Each patient or authorized family member of the patient had signed a written informed consent and agreed to participate in this study. As shown in Fig. 1, 19 liver failure patients suspected with HLH at admission were treated. Eleven patients with a confirmed diagnosis of liver failure and HLH were identified at Beijing You-An Hospital, Capital Medical University, Beijing China, from October 2009 through May 2018. Patients’ demographic, etiologic, clinical, laboratory and therapeutic data were retrieved from medical records. Patients were included in this study of: (1) met the 2012 Chinese Guideline Criteria for diagnosis and treatment of liver failure^1^; (2) did not meet the criteria for diagnosis of HLH (HLH-2004)^10^ at admission but met the criteria during the course of treatment.

### Laboratory variables

A chemistry analyzer (Siemens, ADVIA 2400, Siemens, Germany) was used to measure serum biochemical profiles. Automatic blood cell analyzer (Sysmex, XE-5000) was used to measure peripheral serum components. Automatic Coagulation Analyzer (ACL TOP, IL Corp, America) was used to measure INR and fibrinogen. NK cell activity, CD25 activity and gene detection of HLH were assessed by the Chinese HUA DA gene company.

### Statistical analysis

Statistical analysis was performed using SPSS software (version 13.0, SPSS Corp). Descriptive data were presented as the mean ± SD or median and interquartile range for continuous outcomes; whereas number and percentage (%) for categorical outcomes. Paired-sample T test was used to compare the changes in liver function and whole blood cell counts at diagnosis of liver failure after admission with the earliest definitive diagnosis of HLH. Time-to-event analyses for risk of death were performed with Kaplan-Meier curves. A 2-tailed P < 0.05 was considered significant.

## Result

### Demographic, etiology, types of liver failure and underlying diseases

Eight patients were referred from their primary hospitals, and 3 patients were admitted from our outpatient department, with a median age of 33 years (range: 14~72), including 9 (81.82%) females (Table 1). Types of liver failure and possible etiology were as follows: 1 case of acute-on-chronic liver failure (HBV virus rebound after cessation of Entecavir treatment for 4 months); 2 cases of acute liver failure (1 CMV infection and 1 septic shock); and 8 cases of subacute liver failure (4 cases of drug-induced and 4 of unknown etiology). The main group of underlying diseases consisted of 1 case of cirrhosis with HBV infection and type 2 diabetes, 1 case of systemic lupus erythematosus, and 1 case of pneumonia secondary to ARDS by mechanical ventilation. Seven (63.64%) patients had no identified underlying diseases.

**Table 1:** Demographic/clinical characteristics and prognosis of liver failure with HLH.

Patient ID	Sex (M/F)	Age (years)	Type of liver failure	Time from onset to diagnosis of liver failure (days)	Time from the earliest diagnosis of liver failure to a definitive diagnosis of HLH (days)	MELD score	SOFA score	Vital status	Follow up (days) (up to Dec 2018)
1	F	23	SALF	26	28	12	7	S	2765
2	M	55	ALF	15	24	17	11	D	13
3	F	72	SCALF	17	8	28	8	D	8
4	F	34	SALF	24	18	10	10	D	24
5	F	21	SALF	17	19	12	7	S	3525
6	F	14	ALF	20	8	7	10	D	27
7	F	29	ALF	18	12	12	12	D	23
8	F	24	SALF	28	6	14	7	S	1125
9	F	27	SALF	18	13	6	6	S	1185
10	M	27	SALF	26	32	12	9	S	665
11	F	36	SALF	15	22	11	7	D	18

Abbreviations: F, female; M, male; UK, unknown; CMV, cytomegalovirus; ALF, acute liver failure; SALF, subacute liver failure; ACLF, acute-on-chronic liver failure; MELD score, Model for end-stage liver disease; SOFA score, sequential organ failure assessment score; S, survived; D, death.

### Characteristics of whole blood cells and liver function at diagnosis of HLH

The average interval between onset of disease and diagnosis of liver failure was 20.36 days. The average time from suspected HLH to confirmed HLH was 9.81 days. The average time from the earliest diagnosis of liver failure to a definitive diagnosis of HLH was 17.27 days. The laboratory findings of patients at diagnosis of liver failure and HLH are presented in Table 2. From diagnosis of liver failure after admission to the earliest definitive diagnosis of HLH, the values for white blood cell count (6.74 ± 4.38 vs. 4.79 ± 4.66; p = 0.243), hemoglobin (110.09 ± 18.89 vs. 71.00 ± 13.05; p < 0.001), platelet count (113.45 ± 66.04 vs. 46.73 ± 24.77; p = 0.013), total bilirubin (395.35 ± 108.69 vs. 238.55 ± 129.35; p = 0.002), and INR (1.79 ± 0.22 vs. 1.26 ± 0.21; p < 0.001) were dynamically changed. This finding suggests that HLH can occur in intermediate or late stage of liver failure.

**Table 2:** Comparison of whole blood cell and liver function in patients diagnosed with liver failure after admission in our hospital with the earliest definitive diagnosis of HLH.

Liver failure diagnosed at admission								HLH definitively diagnosed during hospitalization						
Patient ID	WBC1	HGB1	PLT1	ALT1	AST1	TBIL1	INR1	WBC2	HGB2	PLT2	ALT2	AST2	TBIL2	INR2
	×10^9^/L	g/L	×10^9^/L	u/L	u/L	umol/L		×10^9^/L	g/L	×10^9^/L	u/L	u/L	umol/L	
1	1.84	84	114	1341	1066	333	2.16	2.15	86	76	179.2	315.3	95	1.46
2	2.64	107	55	1727.7	706	372.5	1.99	0.56	86	55	146.8	148.9	207	1.62
3	9.26	124	69	620	675	505	1.67	4.33	61	21	64.8	71.3	517	1.33
4	12.12	98	149	679.4	571.1	348.5	2.02	2.75	62	7	145.7	86.4	201	1.12
5	3.85	105	133	203	407	391	2.05	2.22	79	56	52.9	94.8	217	1.36
6	9.32	112	256	409	200.7	445.2	1.52	4.19	72	16	174.7	132.4	176.1	0.84
7	11.04	105	40	739	660	512	1.63	4.3	57	41	279.9	228.7	297	1.07
8	13.72	128	180	823	745.2	402.3	1.83	12.34	89	67	616.9	367.2	349	1.34
9	3.45	99	89	318	436	130.8	1.67	2.77	69	63	96.8	150.5	107.9	1.29
10	4.27	153	125	260	381	510.9	1.66	15.28	71	79	21	50.5	107	1.12
11	2.64	96	38	90.7	358.5	397.6	1.54	1.76	49	33	43.1	164.3	350	1.33

Abbreviations: WBC, white blood cells; Hb, hemoglobin; PLT, platelet; ALT, alanine aminotransferase; AST, aspartate aminotransferase; TBIL, total bilirubin, INR, international normalized ratio.

Reference range: WBC: 3.5–9.5 × 10^9^/L, HGB: 130–175 g/L; PLT: 125–350 × 10^9^/L; Alt: 7–40 u/L (F), 9–50 u/L (M); Ast: 13–35 u/L (F), 15–40 u/L (M); TBIL: 5–21 umol /L; INR: 0.8–1.2.

### Clinical and laboratory characteristics

The clinical features and laboratory findings of patients were presented in Table 3. All patients had experienced severe gastrointestinal symptoms, including fatigue, loss of appetite, abdominal distension, progressive deep jaundice (with an average total bilirubin of 395.35 umol/L) and coagulopathy (with an average INR of 1.79). Clinical and laboratory evaluation was consistent with liver failure. Upon admission, 3 patients had persistent high-grade fever (≥38.5 °C). Fever disappeared upon anti-infection treatment, but recurred in the course of liver failure treatment. The other 8 patients were initially admitted to the hospital without fever but developed high fever during liver failure treatment or even during liver function recovery.

**Table 3:** Clinical andlaboratory findings associated with hemophilia in liver failure with HLH.

Patient ID	Fever (≥38.5 °C)	Splenomegaly	Bone marrow puncture-biopsy	Triglyceride (mmol/l)	Fibrinogen (g/d)	Ferritin (ng/ml)	NK cell activity (%)	sCD25 activity (pg/ml)
1	Y	Y	1+	9.75	0.51	>2000	21.35	46000
2	Y	Y	1−	3.08	0.5	>2000	20.12	32900
3	Y	Y	1−, 2+	1.52	0.9	>2000	30.13	42800
4	Y	Y	1−, 2−, 3+	2.48	1.2	1378	21.12	32000
5	Y	Y	1+	0.93	0.94	455.3	18.23	38000
6	Y	Y	1−, 2+	3.51	0.88	>2000	1875	37200
7	Y	Y	1+	2.36	0.77	>2000	ND	ND
8	Y	Y	1+	1.87	1.69	>2000	12.7	23000
9	Y	Y	1−, 2+	3.77	0.67	>2000	13.79	2882
10	Y	Y	1−, 2+	1.98	1.51	1163	9.75	25000
11	Y	Y	1+	7.04	0.53	>2000	ND	ND

Abbreviations: **Y**, yes; Times of bone marrow: 1+: the first positive, 1-: the first negative, 2+: the second positive, 3+: the third positive;

Reference range: Triglycerides: normal <1.7 mmol/L; Fibrinogen: 2–4 g/L: Ferritin: 30–400 ng/ml (M), 13–150 ng/ml (F); NK cell activity (%): normal ≥15.11%; sCD25 activity: normal <6400 pg/ml; ND, not determined.

During the course of illness, workups for fever included blood, urine and sputum culture, as well as CT examination of the abdomen and chest. The main infectious sites involved abdominal bacterial infection in 5 (45.45%), bacterial pneumonia in 4 (36.36%), fungal pneumonia in 1 (9.09%), and fungal infection in digestive system in 1 (9.09%), and biliary system infection in 4 (36.36%) patients. Six (54.54%) patients presented infection caused by 2 different microorganisms at multiple sites. Brucella, tuberculosis, Rickettsia, parasites and other infections were excluded by laboratory tests. Specific anti-infective agents were administered according to standard therapeutic recommendations from the department of internal medicine. Several patients were unresponsive to empiric antibiotics.

After admission, when liver failure was diagnosed, pancytopenia was not significantly decreased, however, thrombocytopenia occurred early (with platelet counts <100 × 109/L, accounting for 45.45%). During the course of illness, all 11 (100%) patients developed pancytopenia (with an average WBC count of 4.79 × 109/L, an average Hb level of 71.00 g/L and average platelets of 46.73 × 109/L) and cytopenia in >2 blood cell lines (Table 2). Two severe agranulocytosis cases were also observed. For patients with persistent high-grade fever, splenomegaly and pancytopenia, bone marrow biopsy was performed, revealing evidence of hemophagocytosis (histiocytosis, 100%). Further examination for HLH revealed the most frequent laboratory abnormalities as follows: increased ferritin levels (>500 ug/L) in 10 of 11 (90.91%), increased hypertriglyceridemia (>265 mg/dL) in 5 of 11 (45.45%), and decreased hypofibrinogenemia (<1.5 g/L) in 9 of 11 (81.82%). Soluble IL-2 receptor (CD25) levels were increased in 8 of 9 (88.89%), and NK-cell activity was absent or reduced in 3 of 9 (33.33%). These patients met least 5 of the 8 features of the diagnostic criteria for HLH (Table 3) .

### Possible triggers for HLH and organ involvement of liver failure with HLH

The possible triggers for HLH were as follows: bacterial/fungi infections (6/11, 54.55%), drugs (3/11, 27.27%), immune disorders (2/11, 18.18%), CMV infection (1/11, 9.09%), or unknown (2/11, 18.18%).

Liver failure with HLH is a complicated and fatal disease associated with multiple-organ failure. In our study, 3 (27.27%) patients developed renal failure, 5 (45.45%) comorbid with pneumonia and 2 (18.18%) with ARDS. Three (27.27%) patients with maculopapular rash were cured with methylprednisolone. In addition, 5 (45.45%) patients experienced different grades of disturbance of consciousness during disease treatment. Specifically, 4 of 5 patients were accompanied with coma, and 1 of 2 patients was accompanied with ARDS at the time of admission.

### Treatment and prognosis for liver failure with HLH

In our study, liver failure was treated with injection of a glycyrrhizin compound, glutathione, ademetionine, and silymarin. Antibacterial drugs such as imipenem-cilastatin. Cefotaxime Sodium were used to control infections. Transfusion of RBCs or platelet was administered to 3 (27.27%) patients. Two (18.18%) cases underwent plasma exchange, 2 (18.18%) underwent hemofiltration and another 2 (18.18%) underwent mechanical ventilation. Five (45.45%) patients were treated with glucocorticoids to attenuate liver failure before the diagnosis of HLH. The dose of methylprednisolone was 1–1.5 mg/kg per day for 3–5 days, with the longest dosing period for 7 days. The dose of glucocorticoids for liver failure was lower than that for HLH. Three of 5 patients exhibited significant improvement in liver failure. After diagnosis of HLH, the specific therapies for liver failure complicated HLH included glucocorticoids in 11 (100%) patients, or glucocorticoids in combination with 150 mg/m^2^ etoposide weekly in 2 (18.18%) patients, 50 mg/d fludarabine in 2 (18.18%) patients and 20–30 g/d gamma globulin in 5 (45.45%) patients.

Five (45.45%) patients were treated with HLH protocol consisting of dexamethasone (two weekly tapering starting from 10 mg/m^2^) and etoposide (150 mg/m^2^). Regarding the clinical status of patients at diagnosis of HLH, 6 (54.55%) were treated with methylprednisolone at 1–1.5 mg/kg per day, with the dosage being gradually reduced. Each patient was administered with an individualized course of glucocorticoid maintenance, mainly adjusting the dose and time period according to the patient’s clinical indicators.

The MELD and SOFA scores at the earliest definitive diagnosis of HLH and prognosis were listed in Table 1. After follow-up of 17 months (range, 1–142 months), 5 cases (45.45%) survived. Death was attributed to septic shock (2/11), multiple organ failure (3/11), or unknown cause (1/11). The survival rate of 11 patients at 30, 60, and 90 days was 45.45%, 45.45%, and 45.45%, respectively.

## Discussion

HLH, involved multiorgan and multisystem, exhibits progressive aggravation with immune and macrophage dysfunction. Liver failure is often combined with fever, infection, and coagulopathy. How to identify and diagnose HLH early during the treatment of liver failure are essential due to lack of specific clinical manifestations and laboratory findings. Our current knowledge of HLH focuses mainly on clinical aspects. The average time from earliest diagnosis of liver failure to a definitive diagnosis of HLH was 17.27 days in our study. This finding suggests that HLH occurs late in liver failure, specifically within 5 weeks after diagnosis of liver failure. Most of previous reports propose that acute liver failure is attributed to HLH-induced injury^9,11^. To date, detailed mechanisms for HLH-mediated liver injury remain unknown. Probably, infiltration of activated hemophagocytic histiocytes or overproduction of cytokines in HLH can cause damage to one organ or multiple organs, especially liver injury^12^. Alternatively, liver injury can result from underlying diseases. We retrospectively summarized 11 patients diagnosed with liver failure, who did not meet the criteria for diagnosis of HLH (HLH- 2004) on admission, but met the criteria during the course of treatment for liver failure. Based on dynamic changes in liver function, HLH mostly occurs in the middle and late stages, and even during recovery of liver failure,.as demonstrated by decreased bilirubin and INR (Table 2). Thus, HLH can occur during the treatment of liver failure, lag behind liver failure or develop during the recovery of liver failure.

Patients with liver failure are prone to various infections, which act as a trigger for HLH development and a key independent factor for survival in adults with HLH^13^. Therefore, HLH may be associated with complex infections and immune imbalances in liver failure, which is not a manifestation of HLH directly induced damage to organs. In particular, some patients were diagnosed with HLH in the recovery phase of liver failure. This result is similar to that reported by Elia Apodaca *et al*., indicating that HLH is not typically observed in the first days or even weeks. Rather, HLH is diagnosed at a later stage when the patients have already suffered from a number of complications and multiple-organ failure^14^.

Fever in HLH is typically prolonged and unresponsive to antibiotics as accompanied by infections. During the treatment for liver failure, 11 patients (100%) had persistent high-grade fever (≥38.5 °C), splenomegaly and pancytopenia. They were unresponsive to empiric or specific antibiotics at the time of HLH diagnosis; and 3 of 11 (36.36%) patients had no identifiable infectious sites. The presence of hemophagocytes in bone marrow, spleen or lymph nodes is one of the important conditions for HPS. Five (45.45%) patients did not exhibit hemophagocytes in early stage of the disease. Therefore, repeated bone marrow puncture biopsy is necessary in highly suspected cases. Hyperferritinemia is a hallmark of HLH. Serum ferritin levels were increased by ≥500 μg/L in 10 of 11 (90.91%) whereas ≥2000 μg/L 8 of 11 (72.73%) patients. Progressive hyperferritinemia (>2000 ng/mL) in a febrile patient without obvious defects in iron metabolism or transfusion history should prompt analyses of other criteria for HLH^15^. Triglyceridemia and Hypofibrinogenemia are important markers for HLH in adults. Hypertriglyceridemia (>265 mg/dL) was worsened in 5 of 11 (45.45%) patients whereas hypofibrinogenemia (<1.5 g/L) lessened in 9 of 11 (81.82%) patients. The incidence of fibrinogen reduction may be associated with a coagulation disorder caused by liver failure. Soluble IL-2 receptor (CD25) levels were increased in 8 of 9 (88.89%) patients, while NK-cell activity was absent or reduced in 3 of 9 (33.33%) patients.

Optimal treatment of HLH is debated, while the current treatment of HPS is mainly based on HLH-2004 regimen^10^. Induction therapy occurs during the first to eight weeks, and the basic regimen involves dexamethasone, etoposide and cyclosporine. Then, maintenance therapy is administered for 9–40 weeks. Secondary HLH does not require maintenance therapy. Glucocorticoids is a first-line choice for liver failure with HLH, however, optimal dose beneficial for disease control is worth exploring. In our study, 5 (45.45%) patients were treated with glucocorticoids for liver failure before the diagnosis of HLH. Given the early use of glucocorticoids, onset and diagnosis of HLH was likely delayed. Patients with liver failure are prone to various infections, and unable to tolerate the adverse effects of glucocorticoids. If large amounts of corticosteroids are used over a long period of time, secondary infections will become a major cause of death. In our study, 3 of 11 patients died of severe secondary infection in response to high doses of glucocorticoids, representing a life-threatening treatment-related adverse event. Thus, patients with liver failure and HLH can be treated with an initial dose of 1–1.5 mg/kg/d methylprednisolone via intravenous infusion. Then, the dose of methylprednisolone should be reduced gradually every 3–5 days. Our recommended dose of hormone is significantly lower than that in HLH-2004 regimen. Consequently, controlled temperature, improved liver function, hemogram, and clinical manifestation, whereas decreased serum ferritin indicates an effective treatment. According to the patient’s underlying diseases and HLH trigger factors, some patients were administered with gradually reduced or quickly stopped dosage of glucocorticoids.

In previous reports, the prognosis of adult HLH is generally poor, with high early mortality rates ranging from 20 to 44%^16,17^. Zaher K. Otrock *et al*. reported 30-day mortality rate of 30% for adult HLH^18^. In our study, the mortality is approximately 54.55%. The survival rate at 30, 60, and 90 days was 45.45%, 45.45%, and 45.45%, respectively. Dismal prognosis is partially attributed to delayed diagnosis caused by nonspecific HLH clinical manifestations and an overlap with other disease entities and syndromes, such as sepsis and SIRS^19,20^.

Despite the small sample size and retrospective nature of this study, we have demonstrated clinical features of liver failure with HLH, and highlighted the importance of HLH as a differential diagnosis of prolonged fever, jaundice and pancytopenia. This information may provide reference for early diagnosis and treatment of HLH during the course of liver failure.

## Supplementary information

Clinical characteristics of liver failure with hemophagocytic lymphohistiocytosis

## References

[1] Liver Failure and Artificial Liver Group, Chinese Society of Infectious Diseases, Chinese Medical Association; Severe Liver Diseases and Artificial Liver Group, Chinese Society of Hepatology, Chinese Medical Association. Guidelines for diagnosis and treatment liver failure. *Chin J Clin Infect Dis*, **5**(6), 321–327, http://dx.chinadoi.cn/10.3760%2fcma.j.issn.1673-4149.2013.01.001 (2012).

[2] George Melissa R (2014). Hemophagocytic lymphohistiocytosis: review of etiologies and management. J Blood Med.

[3] Li Fei. YY (2015). Clinical characteristics and prognostic factors of adult hemophagocytic syndrome patients: a retrospective study of increasing awareness of a disease from a single-center in China. Orphanet J Rare Dis.

[4] Canna Scott W, Behrens Edward M (2012). Making sense of the cytokine storm: a conceptual framework for understanding, diagnosing, and treating hemophagocytic syndromes. Pediatr. Clin. North Am.

[5] Rosado Flavia GN, Kim Annette S (2013). Hemophagocytic lymphohistiocytosis: an update on diagnosis and pathogenesis. Am. J. Clin. Pathol..

[6] Luísa Azevedo GR, Joana S, Germano I (2015). The challenging diagnosis of haemophagocytic lymphohistiocytosis in an HIV-infected patient. BMJ Case Rep.

[7] Li Jing WQ (2014). Hemophagocytic lymphohistiocytosis: clinical analysis of 103 adult patients. Medicine (Baltimore).

[8] Mustafa Ali M, Ruano Mendez AL, Carraway HE (2017). Hemophagocytic lymphohistiocytosis in a patient with Hodgkin lymphoma and concurrent EBV, CMV, and Candida Infections. J Investig Med High Impact Case Rep.

[9] Jagtap Nitin. SM (2017). Hemophagocytic Lymphohistiocytosis Masquerading as Acute Liver Failure: A Single Center Experience.[J]. J Clin Exp Hepatol.

[10] Henter Jan-Inge, Horne AnnaCarin, Aricó Maurizio, Egeler R. Maarten, Filipovich Alexandra H., Imashuku Shinsaku, Ladisch Stephan, McClain Ken, Webb David, Winiarski Jacek, Janka Gritta (2007). HLH-2004: Diagnostic and therapeutic guidelines for hemophagocytic lymphohistiocytosis. Pediatric Blood & Cancer.

[11] Lin Shide. LY (2012). Acute liver failure caused by hemophagocytic lymphohistiocytosis in adults: A case report and review of the literature[J]. Medicine, 2016.

[12] Claire L (2012). Hemophagocytic lymphohistiocytosis in adults: diagnosis and treatment.[J]. Joint Bone Spine.

[13] Brito-Zerón Pilar KB (2018). Prognostic Factors of Death in 151 Adults With Hemophagocytic Syndrome: Etiopathogenically Driven Analysis. [J]. Mayo Clin Proc Innov Qual Outcomes.

[14] Apodaca Elia. R-RS, Juventina T-AE (2018). Demichelis-Gómez Roberta. Prognostic Factors and Outcomes in Adults With Secondary Hemophagocytic Lymphohistiocytosis: A Single-center Experience.[J]. Clin Lymphoma Myeloma Leuk.

[15] Rafał M, Gritta J, Wieslaw W-J (2017). Similar but not the same: Differential diagnosis of HLH and sepsis.[J]. Crit. Rev. Oncol. Hematol.

[16] Parikh Sameer A, Kapoor P, Letendre L, Kumar S, Wolanskyj AP (2014). Prognostic factors and outcomes of adults with hemophagocytic lymphohistiocytosis.[J]. Mayo Clin. Proc..

[17] Arca Marc. FL (2015). Prognostic factors of early death in a cohort of 162 adult haemophagocytic syndrome: impact of triggering disease and early treatment with etoposide.[J]. Br. J. Haematol.

[18] Otrock Zaher K, Grossman Brenda J, Eby Charles S (2018). Transfusion requirements and 30-day mortality predictors for adult hemophagocytic lymphohistiocytosis.[J]. Int. J. Hematol.

[19] Raschke Robert A, Garcia-Orr R (2011). Hemophagocytic lymphohistiocytosis: a potentially underrecognized association with systemic inflammatory response syndrome, severe sepsis, and septic shock in adults.[J]. Chest.

[20] Padhi Somanath. VRG’B, Ramdas A, Phansalkar MD, Sarangi R (2013). Hemophagocytic lymphohistiocytosis: critical reappraisal of a potentially under-recognized condition.[J]. Front Med.

