# Supplementary material for: Clinical characteristics of liver failure with hemophagocytic lymphohistiocytosis
Source: Sci Rep. 2019 May 31;9:8125. doi: 10.1038/s41598-019-43909-w (PMC6544643; doi:10.1038/s41598-019-43909-w)
Supplement: Supplementary file 1 — Clinical characteristics of liver failure with hemophagocytic lymphohistiocytosis [file 41598_2019_43909_MOESM1_ESM.pdf]

Clinical characteristics of liver failure with hemophagocytic lymphohistiocytosis

Jinling Dong[1,#], Fang Xie[1,#], Lin Jia[1], Juan Li[1], Zhongjie Hu[1], Yueke Zhu[1], Hongwei Yu[1], Yujuan Zhao[2], Qinwei Yao[1], Qinghua Meng[1,*]

The criteria for diagnosis of liver failure with HLH：

The criteria for diagnosis and treatment of liver failure were based on the 2012 Chinese Guideline Criteria. The clinical diagnosis of liver failure is according to a comprehensive analysis of medical history, clinical manifestations, and auxiliary examinations as outlined below:

(1) Acute liver failure: acute onset, development of hepatic encephalopathy within 2 weeks (divided by four-degree classification) with manifestations: a. extreme fatigue, obvious anorexia, bloating, nausea, vomiting, and severe gastrointestinal symptoms; b. short-term jaundice that progressively worsens; c. obvious bleeding tendency, plasma prothrombin activity (PTA) ≤ 40% (or INR ≥ 1.5)without other causes; d. progressive liver dysfunction.

(2) Subacute liver failure: relatively urgent onset, with manifestations in 2~26 weeks: a. extreme fatigue, obvious gastrointestinal symptoms; b. jaundice that rapidly worsens, with serum total bilirubin (TBil) 10-fold greater than the upper limit of normal or daily increase by ≥ 17.1 μmol/L; c.± hepatic encephalopathy; d. obvious bleeding tendency, PTA ≤ 40% (or INR ≥ 1.5) without other causes.

(3) Acute-on-chronic liver failure: On the basis of chronic liver disease, the clinical syndrome of acute or subacute liver function decompensation in a short period of time characterized by: a. extreme fatigue and obvious gastrointestinal symptoms; b. Astragalus that rapidly worsens, with serum TBil > 10-fold greater than the upper limit of normal value or daily increase by ≥17.1 μmol/L; c. bleeding tendency, PTA ≤ 40% (or INR ≥ 1.5) without other causes; d. decompensated ascites; e.± hepatic encephalopathy[1].

The HLH was diagnosed according to the 2004 Histiocyte Society criteria (HLH-2004) when meeting at least five of the following eight criteria: (1) fever ≥ 38.5 °C; (2) splenomegaly; (3) cytopenia affecting at least 2 lineages: hemoglobin < 90 g/L, platelet count < 100 × 10^9/L, absolute neutrophil count (ANC) < 1 .0× 10^9/L; (4) hypertriglyceridemia ≥ 265 mg/dL and/or hypofibrinogenemia ≤ 1.5 g/L; (5) histologic evidence of hemophagocytosis (bone marrow, lymph nodes, spleen, or liver); (6) serum ferritin ≥ 500 mg/L; (7) soluble CD25 (i.e., soluble IL-2 receptor) ≥ 2400 U/mL; and (8) low or absent NK cell activity [10].

The study was conducted according to the Helsinki Declaration and approved by Institutional Review Board of Beijing You-An Hospital.

Consent statement

The study was conducted according to the Helsinki Declaration and approved by Institutional Review Board of Beijing You-An Hospital. Each patient or authorized family member of the patient signed a written informed consent and agreed to participate in the study.
